# Non-invasive thoracoabdominal mapping of postoesophagectomy conduit function

**DOI:** 10.1093/bjsopen/zrad036

**Published:** 2023-05-05

**Authors:** Tim Hsu-Han Wang, Ashraf Tokhi, Armen Gharibans, Nicholas Evennett, Grant Beban, Gabriel Schamberg, Chris Varghese, Stefan Calder, Cuong Duong, Greg O’Grady

**Affiliations:** Department of Surgery, The University of Auckland, Auckland, New Zealand; Division of Cancer Surgery, Peter MacCallum Cancer Centre, Melbourne, Victoria, Australia; Department of Surgery, The University of Auckland, Auckland, New Zealand; Alimetry Ltd, Auckland, New Zealand; Auckland Bioengineering Institute, The University of Auckland, Auckland, New Zealand; Department of Surgery, Auckland City Hospital, Auckland, New Zealand; Department of Surgery, Auckland City Hospital, Auckland, New Zealand; Department of Surgery, The University of Auckland, Auckland, New Zealand; Alimetry Ltd, Auckland, New Zealand; Department of Surgery, The University of Auckland, Auckland, New Zealand; Department of Surgery, The University of Auckland, Auckland, New Zealand; Alimetry Ltd, Auckland, New Zealand; Division of Cancer Surgery, Peter MacCallum Cancer Centre, Melbourne, Victoria, Australia; Department of Surgery, The University of Auckland, Auckland, New Zealand; Alimetry Ltd, Auckland, New Zealand; Auckland Bioengineering Institute, The University of Auckland, Auckland, New Zealand

## Introduction

Oesophagectomy is a complex procedure performed for malignant and benign conditions. Procedural variations exist, dependent on patient and disease factors, with the stomach typically being used for reconstruction. Postoesophagectomy conduit dysfunction is common, including delayed gastric conduit emptying (DGCE) (approximately 30 per cent), gastro-oesophageal reflux disease (approximately 80 per cent) and other chronic symptoms without a mechanical cause^1,2^. Emerging evidence implicates abnormal gastric electrophysiology as a contributing factor^3–5^.

Although conduit dysfunction is multifactorial, dysmotility is a common contributing mechanism. However, it is clinically challenging to distinguish patients with dysmotility as opposed to alternative causes for symptoms (for example obstruction or pyloric dysfunction), as current tests such as endoscopy, fluoroscopy, radionuclear imaging and manometry have limited accuracy, and/or are invasive or involve radiation. A safe and accurate test is needed to reliably assess conduit motility to inform correct therapy.

Gastric Alimetry® (Auckland, New Zealand) is a new non-invasive test to evaluate gastric electrophysiology and function at high resolution, recently receiving regulatory approvals for clinical use^6^. This technique has been extensively validated^3,7,8^ and is being applied in medical disorders, but has yet to be used for postoperative patients. This study therefore evaluated the feasibility of applying Gastric Alimetry after oesophagectomy to assess conduit motility.

## Methods

Patients who underwent oesophagectomy in Auckland, New Zealand, within the last 3 years were invited to participate following ethical approval (AH1125). Patients were excluded if they were undergoing chemotherapy/radiotherapy, had not undergone postoperative computed tomography (CT) or suffered mechanical obstruction. Clinical data including operation notes, imaging, endoscopy and histopathology were evaluated.

**Fig. 1:**
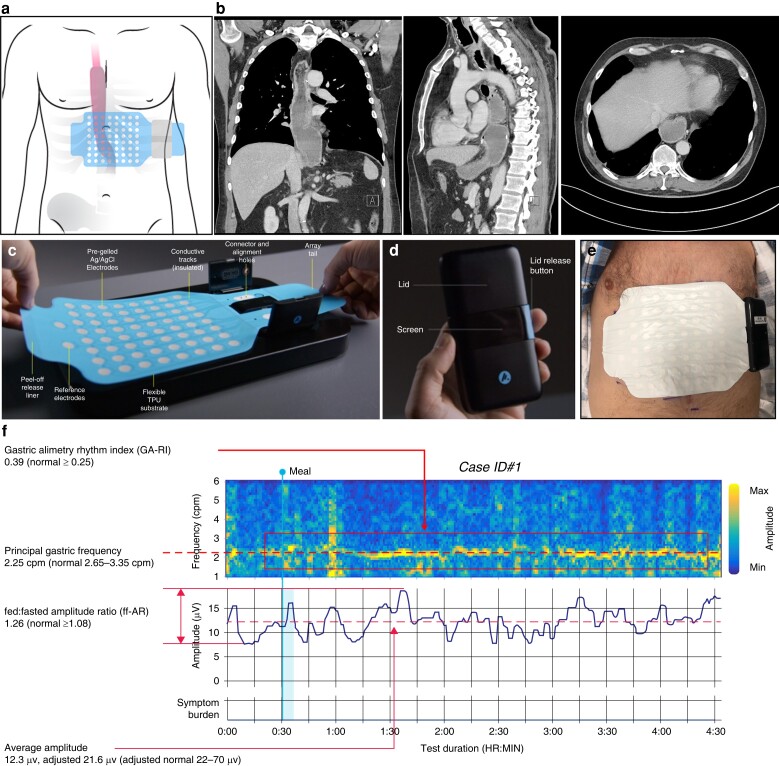
**a** Array placement in relation to the thoracoabdominal gastric conduit. **b** Coronal, sagittal and axial views from CT scans of ID#1 illustrating the location of the stomach located in the posterior mediastinum, behind the heart, lungs and liver. **c** Gastric Alimetry stretchable electrode array. **d** Gastric Alimetry wearable Reader. **e** Gastric Alimetry electrode array and wearable Reader on the patient’s thoracoabdominal region, per the placement depicted in **a. f** Spectral map for Case ID#1 illustrating a reduced principal gastric frequency and BMI-adjusted average amplitude. Patient photo and imaging are used with patient's written consent. AgCl, silver chloride; TPU, thermoplastic polyurethane.

Gastric Alimetry was performed under a protocol adapted for oesophagectomy. This device comprises a high-resolution stretchable electrode array (8 × 8 electrodes; 20 mm spacing; 196 cm^2^), a wearable Reader, validated iOS app for symptom logging and a cloud-based reporting platform (*Fig. 1*)^9–11^. Patients were fasted for >6 h before array placement as guided by gastric position on CT (*Fig. 1a–e*). After a 30 min baseline recording, patients consumed a 218 kcal meal (100 ml nutrient drink and half an oatmeal energy bar), followed by a 4-hour postprandial recording with concurrent symptom logging.

Spectral analysis was performed, encompassing four established metrics^12^: principal gastric frequency, BMI-adjusted amplitude, Gastric Alimetry Rhythm Index (GA-RI; reflecting pacemaker stability), fed:fasted amplitude ratio (ff-AR; indicating meal response with contractions), with comparison to reference intervals^13^. Frequency was not reported if there was no rhythm (as measured by GA-RI)^10^. Adverse events were recorded. Data were evaluated with descriptive statistics.

## Results

Demographic and operative data are reported in *Table S1*. Six patients were recruited (all males; median age 65.5 years; range 58–73). Oesophagectomies were performed between 6.5 months and 3 years prior, with the standard procedure including vagotomy and pyloroplasty. Indications were cancer (*n* = 4), Barrett’s oesophagus (*n* = 1) and achalasia (*n* = 1). One case (ID#6) developed a necrotic gastric conduit prompting resection, formation of cervical oesophagostomy and feeding jejunostomy on day 6 following surgery, with subsequent colonic interposition graft with Roux-en-Y reconstruction 8 months later. This case served as a negative control.

All patients except one were largely asymptomatic at the time of testing. The symptomatic patient (ID#5) reported moderate to severe nausea, vomiting, early satiation, abdominal pain, reflux and a poor quality of life.

**Fig. 2:**
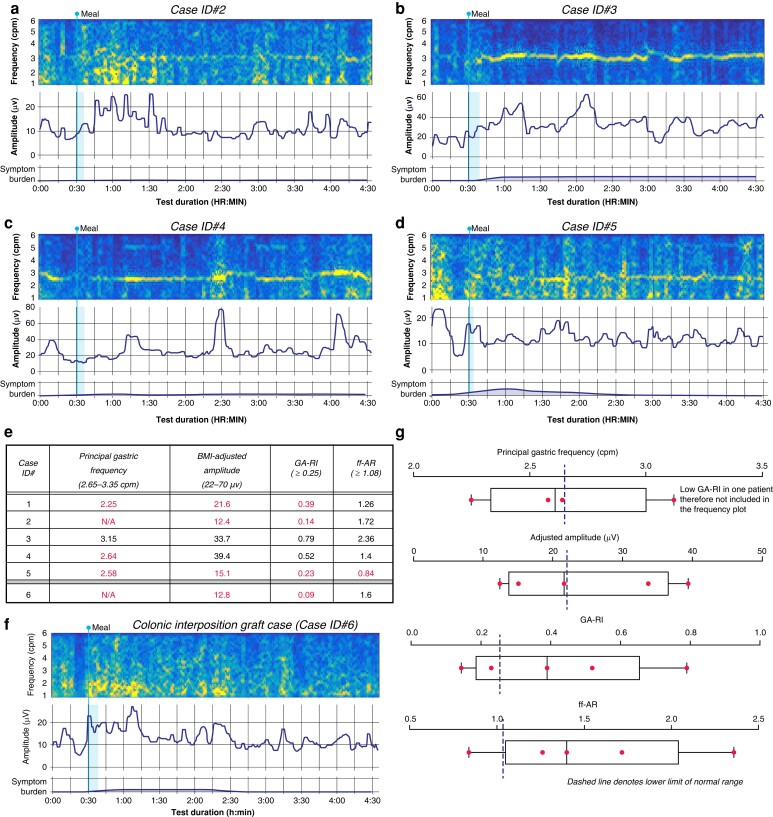
**a–d** illustrates case ID#2 to ID#5’s spectral maps with associated symptom burden plots. Quantitative analysis is presented in **e** with reference intervals as shown. Quantitative results for case ID#1 shown in *Fig. 1f* are also presented in **e**. **f** Spectrogram for the patient with the colonic interposition graft. **g** Box and whiskers graph for the quantitative results for case ID#1 to ID#5. The dashed line represents the lower limit of the reference interval for each Gastric Alimetry spectral metric. ff-AR, fed:fasted amplitude ratio; GA-RI, gastric alimetry rhythm index.

Gastric activity was successfully captured non-invasively in all cases (*Figs. 1*, *2*). Four cases (IDs#1–2, 4–5) had at least one abnormal parameter, all showing reduced motility profiles (*Fig. 1f*, *Fig. 2a, c–e*). Of these, low or abnormal frequency was the most common abnormality (4/4 cases), followed by low amplitude in 3/4, low GA-RI in 2/4 and low ff-AR in 1/4 (*Fig. 2e*). The symptomatic patient (ID#5) was found to have abnormalities in all four domains, with symptoms being maximal when activity was weakest (*Fig. 2d, e*). ID#3 was the only case with normal parameters throughout (*Fig. 2b, e*), who had minimal gastric resection (<4 cm).

In the negative control (ID#6), no gastric activity was identified, but low frequency burst activity was evidence consistent with immediate colonic activity postprandially (*Fig. 2f*)^14^.

No adverse reactions occurred.

## Discussion

Persistent upper gastrointestinal symptoms in the absence of mechanical obstruction are common after oesophagectomy. Contributing factors include conduit dysmotility, hypersensitivity/pain syndromes, dumping syndrome and pyloric dysfunction, which may overlap and are difficult to differentiate on clinical history and current tests. This study shows the safety and feasibility of a new test called Gastric Alimetry for non-invasively evaluating the function of the deep-seated postoesophagectomy gastric conduit.

Gastric surgery modifies the electrical conduction system that coordinates contractions^15^, with previous studies implicating abnormal electrophysiology in conduit dysfunction^3–5^. However, reliable techniques to assess conduit function have been lacking. Recent advances have enabled substantial progress in evaluating gastric electrophysiology in health and disease^7,16,17^. A legacy technique termed electrogastrography (EGG) previously attempted to capture gastric electrical activity from the skin surface, but was limited by low resolution and high sensitivity to noise^5^. Gastric Alimetry overcomes these problems by employing a high-resolution array together with sophisticated signal processing algorithms^9,10^, which were shown to be effective even with conduits positioned in the thorax and posterior mediastinum. The patient with a total gastrectomy and colonic interposition graft served as a negative control, further increasing confidence in the current findings.

Previously, Gastric Alimetry has been exclusively performed in patients with normal gastric anatomies, in whom reference ranges were developed^12,13,17^. Some adjustments to interpretations will therefore be required as the test is applied to postoperative patients. Specifically, normative values for amplitude will need to be redefined due to the greater distance between the stomach and the array, and this work is currently in progress. Additionally, meal sizes were reduced by 50 per cent *versus* the standard Gastric Alimetry test to account for the reduced gastric remnant volume, which is considered adequate to stimulate gastric activity^6^.

Reduced motility parameters were the dominant finding in this post-oesophagectomy cohort, observed as reductions in frequency, rhythm stability (GA-RI) and meal responses (ff-AR), except for one patient who had a minimal gastric resection. Reduced frequency likely reflects resection of the native gastric pacemaker, leading to the development of a new lower frequency pacemaker^18^. Low GA-RI likely reflects gastric neuromuscular dysfunction due to aberrant pacemaker recovery^6^, while reduced meal responses could reflect loss of vagal input^15^. While vagotomy is inevitable to allow lymph node harvest in cancer patients, evolving techniques that offer vagal-sparing oesophagectomies for non-malignant indications (for example achalasia or stricturing disease) may assist in avoiding vagotomy-associated complications^19^.

Validated symptom profiling is also performed with the Gastric Alimetry test. While detailed symptom analysis was not a focus of this feasibility study, symptom profiling is proving useful elsewhere in distinguishing cases with hypersensitivity and pain syndromes, particularly when gastric function is normal, and is likely to be valuable in future postoperative studies^6,11^. Emerging spatial mapping techniques will also allow determination of electrical propagation patterns in future studies^9^.

With feasibility established, future studies can now be conducted applying this technique on larger cohorts of patients after oesophagectomy. Such work will enable improved characterization of pathophysiology and symptom correlations, in order to guide therapeutic decisions, as is being performed in other gastric disorders^6^. In addition, the new test is also now being evaluated for its potential in gastric dysfunction after pancreaticoduodenectomy^20^.

## Supplementary Material

zrad036_Supplementary_Data

## Data Availability

Data from this study have not been entered in a public repository. Instead, the data used in this study are clearly illustrated in the Figures and *Table S1*.
