# Supplementary material for: Non-invasive thoracoabdominal mapping of postoesophagectomy conduit function
Source: BJS Open. 2023 May 5;7(3):zrad036. doi: 10.1093/bjsopen/zrad036 (PMC10162678; doi:10.1093/bjsopen/zrad036)
Supplement: zrad036_Supplementary_Data [file zrad036_supplementary_data.docx]

**Non-invasive thoracoabdominal mapping of post-oesophagectomy conduit function**

Tim Hsu-Han Wang^1^, Ashraf Tokhi^2^, Armen Gharibans^1, 3,4^, Nicholas Evennett^5^, Grant Beban^5^, Gabriel Schamberg^1,3^, Chris Varghese^1^, Stefan Calder^1,3^, Cuong Duong^2^, Greg O’Grady^1, 3,4^

**Affiliations**

1. Department of Surgery, the University of Auckland, New Zealand
2. Division of Cancer Surgery, Peter MacCallum Cancer Centre, Victoria, Australia
3. Alimetry Ltd, Auckland, New Zealand
4. Auckland Bioengineering Institute, The University of Auckland, New Zealand
5. Department of Surgery, Auckland City Hospital, Auckland, New Zealand

**Corresponding Author**

Professor Greg O’Grady

Department of Surgery, University of Auckland, New Zealand

Private Bag 92019, Auckland Mail Centre, Auckland 1142, New Zealand

[greg.ogrady@auckland.ac.nz](mailto:greg.ogrady@auckland.ac.nz)

**Supplementary Materials - Index**

| **Supplementary Results** |  |
| --- | --- |
| Table S1 | *pag. 2* |

**Supplementary Results**

**Table S1**

| ID# | Age (years) | Gender | Procedure | Indication | Amount of stomach resected | BMI | Interval between surgery and mapping (months) |
| --- | --- | --- | --- | --- | --- | --- | --- |
| 1 | 58 | M | Hybrid Ivor-Lewis oesophagectomy and left adrenalectomy | Achalasia | 9.5cm x 4.5cm x 3.5cm of stomach resected | 28 | 14 |
| 2 | 64 | M | Minimally invasive three-stage oesophagectomy | Oesophageal cancer | 14cm greater curvature,  5.5cm lesser curvature | 22.3 | 17 |
| 3 | 69 | M | Hybrid Ivor-Lewis oesophagectomy | Oesophageal cancer | 4cm greater curvature,  4cm lesser curvature | 22.9 | 36 |
| 4 | 63 | M | Minimally invasive oesophagectomy | Barrett's oesophagus with high grade dysplasia | 4.5cm lesser curvature | 30.4 | 6.5 |
| 5 | 67 | M | Minimally invasive three-stage oesophagectomy | Oesophageal cancer | 13cm greater curvature | 23.2 | 15.5 |
|  |  |  |  |  |  |  |  |
| 6 | 73 | M | 1. Hybrid two-stage oesophagectomy 2. Colonic interposition graft | Oesophageal cancer | 20cm greater curvature,  12.5cm lesser curvature | 21.7 | 12 |
